# Triple negative tumors accumulate significantly less methylglyoxal specific adducts than other human breast cancer subtypes

**DOI:** 10.18632/oncotarget.2121

**Published:** 2014-06-20

**Authors:** Barbara Chiavarina, Marie-Julie Nokin, Florence Durieux, Elettra Bianchi, Andrei Turtoi, Olivier Peulen, Paul Peixoto, Philippe Irigaray, Koji Uchida, Dominique Belpomme, Philippe Delvenne, Vincent Castronovo, Akeila Bellahcène

**Affiliations:** ^1^ Metastasis Research Laboratory, GIGA-Cancer, University of Liège, Liège, Belgium; ^2^ Department of Anatomy and Pathology, University of Liège, Liège, Belgium; ^3^ Association for Research and Treatments Against Cancer (ARTAC), Paris, France; ^4^ Laboratory of Food and Biodynamics, Graduate School of Bioagricultural Sciences, Nagoya University, Nagoya, Japan

**Keywords:** methylglyoxal, breast cancer, advanced glycation end-products, Arg-pyrimidine adducts, glyoxalase 1

## Abstract

Metabolic syndrome and type 2 diabetes are associated with increased risk of breast cancer development and progression. Methylglyoxal (MG), a glycolysis by-product, is generated through a non-enzymatic reaction from triose-phosphate intermediates. This dicarbonyl compound is highly reactive and contributes to the accumulation of advanced glycation end products. In this study, we analyzed the accumulation of Arg-pyrimidine, a MG-arginine adduct, in human breast adenocarcinoma and we observed a consistent increase of Arg-pyrimidine in cancer cells when compared with the non-tumoral counterpart. Further immunohistochemical comparative analysis of breast cancer subtypes revealed that triple negative lesions exhibited low accumulation of Arg-pyrimidine compared with other subtypes. Interestingly, the activity of glyoxalase 1 (Glo-1), an enzyme that detoxifies MG, was significantly higher in triple negative than in other subtype lesions, suggesting that these aggressive tumors are able to develop an efficient response against dicarbonyl stress. Using breast cancer cell lines, we substantiated these clinical observations by showing that, in contrast to triple positive, triple negative cells induced Glo-1 expression and activity in response to MG treatment. This is the first report that Arg-pyrimidine adduct accumulation is a consistent event in human breast cancer with a differential detection between triple negative and other breast cancer subtypes.

## INTRODUCTION

Breast cancer represents the highest incidence of cancers and the second most common cause of death in women. This malignancy is characterized by a highly heterogeneous group of lesions with molecular and biochemical signatures, disease course, prognosis and treatment response. The classification based on gene expression patterns is commonly used to assess prognosis and therapy regimen. Breast malignant tumors are categorized into 4 main subtypes: luminal A (or HER2 negative tumors), luminal B (or triple positive tumors), HER2 positive, and basal-like (or triple negative tumors) [1-3]. This latter group represents around 15-20% of newly diagnosed breast cancers [4]. Typically, these lesions are characterized by high proliferation rate, high histologic grade, tumor necrosis and poor prognosis [5, 6]. Currently no treatment except chemoterapy is available for these patients.

Emerging evidence shows that cancer is a metabolic disease and that altered cellular energy metabolism is a common feature of malignant tumors. In recent studies, a link between obesity, diabetes and breast cancer has been made through insulin/insulin-like growth factor and PI3K/Akt/mTOR signaling pathways and through obesity-induced chronic inflammation caused by adipose tissue dysfunction [7-9].

In proliferating cells, glucose metabolism and growth control are strictly linked [10, 11]. The Warburg effect according to which cells can rely on glycolysis as a major source of energy rather than oxidative phosphorylation, even in normoxic conditions, has been well described in many tumors including breast, melanoma, lung and colorectal cancer [12, 13]. Methylglyoxal (MG) is a highly reactive dicarbonyl compound and a potent glycating agent, mainly generated as a by-product of glycolysis through a spontaneous degradation of triosephosphates [14]. Accumulation of glycated proteins has been observed in several human diseases including diabetes, inflammation, aging, neurodegenerative disorders and cancer [15-25].

MG reacts with arginine, lysine and cysteine residues, nucleic acids and lipids generating advanced glycation end products (AGEs). In mammalian cells, MG is detoxified by the glyoxalase system, an enzymatic pathway consisting of two enzymes called glyoxalase 1 (Glo-1) and glyoxalase 2 (Glo-2). Glo-1 catalyzes the isomerisation of the hemithioacetal, formed spontaneously from MG and reduced glutathione (GSH) to S-D-lactoylglutathione. The second enzyme, Glo-2, converts the S-D-lactoylglutathione to D-lactate and recycles reduced glutathione [26, 27]. It has been shown that Glo-1 expression and activity is increased in many human cancer types such as colon [28], prostate [29, 30], melanoma [31], lung [32], and breast [33] and that Glo-1 overexpression is correlated with cancer progression and drug resistance [34, 35]. In a recent proteomic study, Glo-1 expression has been shown to be positively correlated with high tumor grade in breast cancer [36].

The reaction of MG with arginine residues generates stable MG-moieties called Arg-pyrimidine [37, 38]. So far only few studies analyzed the expression of Arg-pyrimidine adducts in cancer. Van Heijst and collaborators performed an Arg-pyrimidine immunohistochemical evaluation in 4 different types of human cancers, including a limited number of breast cancer lesions (n=5). Their conclusion was that Arg-pyrimidine level differs greatly between different types of tumors. To date the best-studied Arg-pyrimidine-modified protein identified in malignant tumors is the heat shock protein 27 (Hsp27). This chaperone protein facilitates the proper refolding of damaged proteins and plays a key role in cell resistance to stress. In cancer cells, MG post-translationally modified Hsp27 prevented apoptotic cell death notably through the inhibition of cytochrome C-mediated caspase activation [39-41].

In this study, we evaluated for the first time Arg-pyrimidine adducts accumulation in a large collection of breast cancer lesions categorized into the 4 main molecular subtypes using an immunohistochemistry approach. We demonstrated that triple negative breast tumors accumulate least Arg-pyrimidine adducts because of an efficient Glo-1-based detoxification system. These findings suggest that sensitizing triple negative tumors to carbonyl stress may represent a good strategy for aggressive cancer subtypes.

## RESULTS

### Arg-pyrimidine adducts are accumulated in human breast cancer tissues

**Figure 1:**
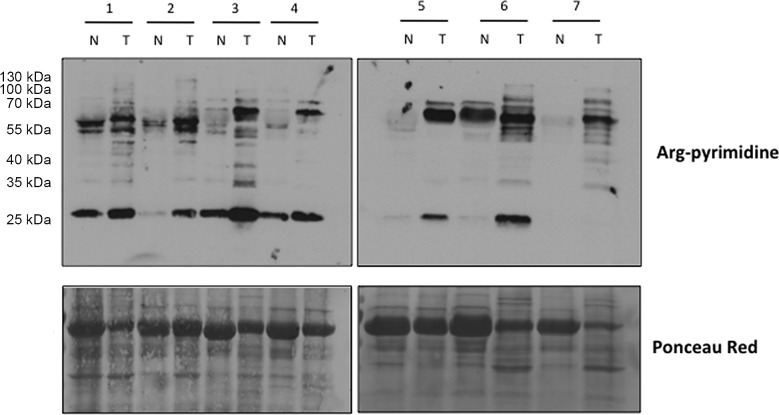
Increased Arg-pyrimidine residues in human breast cancer tissues compared with the non-tumoral counterparts Arg-pyrimidine adducts were evaluated in 7 breast cancer tissues (T) and in their non-tumoral counterpart (N) using western blot analysis (20 μg of proteins were loaded). The Ponceau S-stained membrane is shown as an indicator of equal protein loading.

We evaluated the presence of Arg-pyrimidine adducts in 7 breast cancer tissues and in their non-tumoral counterparts by Western blot analysis. The antibody was specifically directed against Arg-pyrimidine adducts. As shown in Figure 1, tumoral lesions accumulated more Arg-pyrimidine moieties in comparison with the corresponding non-tumoral counterparts. Some MG-modified proteins appeared enriched and/or uniquely observed in cancer lesions.

### Triple negative breast tumors accumulate less Arg-pyrimidine adducts compared with other breast cancer subtypes

**Figure 2:**
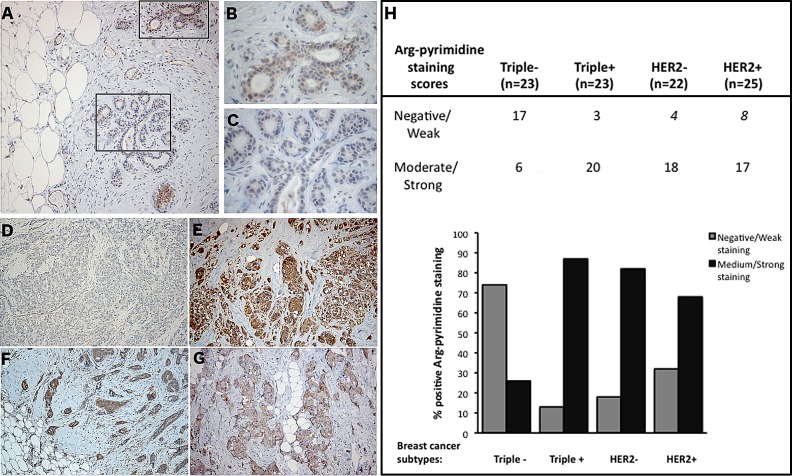
Triple negative breast tumors accumulated less Arg-pyrimidine adducts in comparison with triple positive, HER2− and HER2+ tumors (A) Arg-pyrimidine adducts are detectable in breast tissue from reduction mammoplasty. The panels (B) and (C) show a higher magnification (400x) of the cellular regions boxed in panel A. Triple negative lesions (D) exhibited a significantly lower accumulation of Arg-pyrimidine residues compared to triple positive (E), HER2− (F) and HER2+ (G) subtypes, magnification 100x. Immunohistochemical quantification (H) was performed as described in Materials and Methods section.

Using immunohistochemistry, we next examined Arg-pyrimidine accumulation in 2 normal human breast samples and in a collection of 93 breast tumors categorized in triple negative, triple positive, HER2 positive and HER2 negative. Arg-pyrimidine staining was mainly localized to the cytoplasm of breast epithelial cells, however in some cells a nuclear staining was also detectable. In good accordance with the Western blot analysis conducted on adjacent non tumoral breast samples, a moderate Arg-pyrimidine immunostaining was detected in normal breast tissue (Figure 2A-C). It is noteworthy that endothelial cells lining the visible blood vessels adjacent to normal mammary glands were positive (Figure 2A). Interestingly, when we evaluated Arg-pyrimidine accumulation in the different breast cancer subtypes we found that Arg-pyrimidine staining was significantly associated (chi-square test p<0.0001) with the cancer subtypes. Indeed, we observed that the majority of triple negative lesions (Figure 2D) exhibited low staining for MG-adducts in comparison with the other subtypes (Figure 2E-G). In fact, more than 70% of triple negative breast tumors exhibited a negative to weak staining for Arg-pyrimidine while the majority of the 3 other breast cancer subtypes evaluated showed a moderate to strong level of MG-adducts (Figure 2H). No significant staining was observed in tumor stroma in all the sections analyzed.

### Triple negative breast cancer cell lines globally showed less Arg-pyrimidine adducts than other tumor subtypes

**Figure 3:**
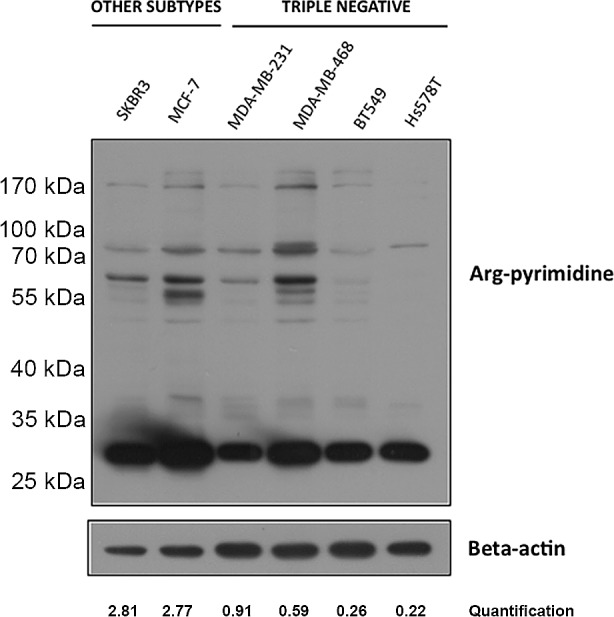
Low basal Arg-pyrimidine adducts accumulation in triple negative breast cancer cell lines Arg-pyrimidine levels were evaluated using Western blot analysis on 6 breast cancer cell lines including SKBR3, MCF-7 and 4 triple negative cell lines. In comparison with MCF-7 and SKBR3, all triple negative cells expressed less Arg-pyrimidine accumulation. Beta-actin expression was used to normalize the amount of loaded proteins. Semi-quantitation of total Arg-pyrimidine adducts was done using beta-actin.

Next, we investigated Arg-pyrimidine accumulation in HER2 positive (SKBR3), estrogen/progesterone receptor-positive (MCF-7) and 4 triple negative (MDA-MB-231, MDA-MB-468, BT549, Hs579T) human breast cancer cell lines using Western blot analysis. All triple negative cell lines showed less Arg-pyrimidine accumulation compared with non-triple negative cell lines (Figure 3). This result is consistent with our immunohistochemical observations indicating that different breast cancer subtypes show distinct glycated adducts accumulation.

### Increased glyoxalase 1 (Glo-1) activity in triple negative tissues

**Figure 4:**
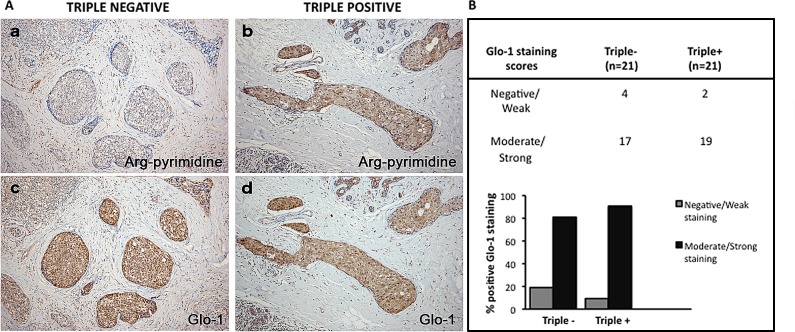
Representative immunohistochemical staining of Glo-1 expression in triple negative versus triple positive human breast tissues Glo-1 expression was evaluated in triple negative (n= 21) and triple positive (n= 21) breast cancer patients and one representative case of each is shown in panel A (c, d). The staining of Glo-1 was compared with the immunostaining for Arg-pyrimidine accumulation in the same triple negative (a) and triple positive (b) human specimen. Glo-1 is highly expressed in both triple positive and triple negative tumors and there was no significant difference between the two breast cancer subtypes. Glo-1 immunohistochemical quantification is shown in panel (B). The evaluation of the staining was performed as described in Materials and Methods section. Magnification 100x.

**Figure 5:**
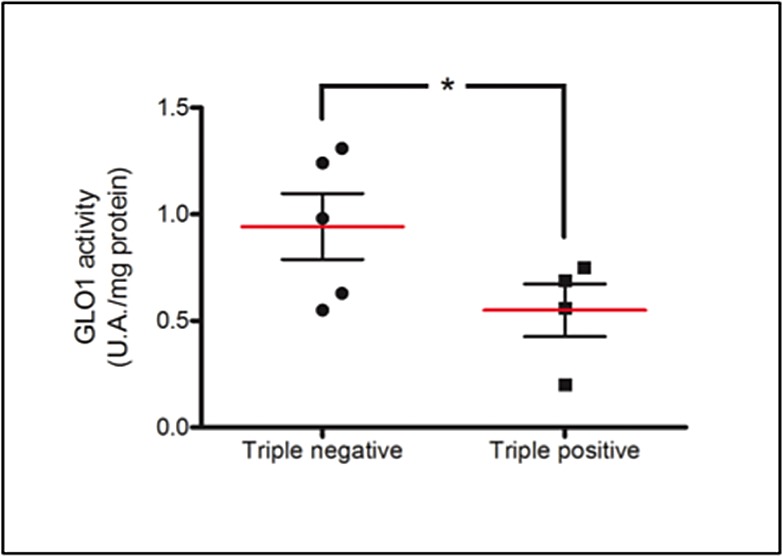
Increased Glo-1 activity in triple negative breast cancer tumors in comparison with triple positive ones Glo-1 activity was measured in breast cancer tumor samples comprising 4 triple positive and 5 triple negative lesions. Glo-1 enzymatic assay was performed on tissue total protein extracts as described in Material and Methods. Statistical comparison was performed by one-tailed Student's t-test (*p ≤ 0.05).

To further explore the observed difference in accumulation of glycated adducts, serial sections were stained for both Arg-pyrimidine (Figure 4A, a and b) and the main MG-detoxifying enzyme, Glo-1 (Figure 4A, c and d). High Glo-1 staining was observable in both tumor subtypes regardless of the different levels of Arg-pyrimidine and no significant association (Fisher's exact test) was observed between the staining intensity and the different cancer subtypes evaluated. Indeed, most of triple negative (80%) and triple positive (90%) lesions expressed high levels of Glo-1 (Figure 4B). Therefore, we sought to examine the enzymatic activity of Glo-1 in these samples. The measurement performed on tissue extracts showed a significant increase in Glo-1 activity in triple negative tumors when compared with triple positive ones (Figure 5).

### Only triple negative breast cancer cell lines respond to MG stimulation by increasing their Glo-1 expression and activity

**Figure 6:**
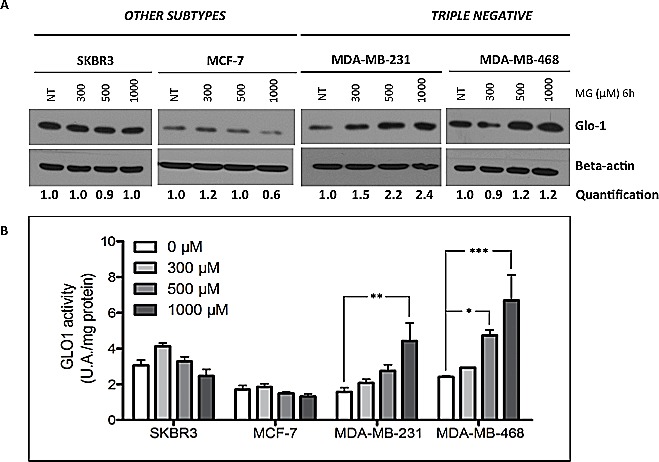
Glo-1 expression and activity in human breast cancer cells upon MG stimulation Breast cancer cells were treated with MG at 300, 500 or 1000 μM for 6 hours. Only triple negative cancer cells are capable to respond to MG treatment by up-regulating both Glo-1 expression (A) and activity (B). No modulation of Glo-1 was observed in the other cell subtypes analyzed. Beta-actin expression was used to normalize the amount of loaded proteins. Semi-quantitation analysis was done using beta-actin as a loading control. Results are the average of independent experiments (n= 3). Data were statistically analyzed with two-way ANOVA followed by Bonferroni multiple comparisons (* *p* ≤ 0.05, **p ≤ 0.01 and ***p ≤ 0.001 vs untreated cells).

**Figure 7:**
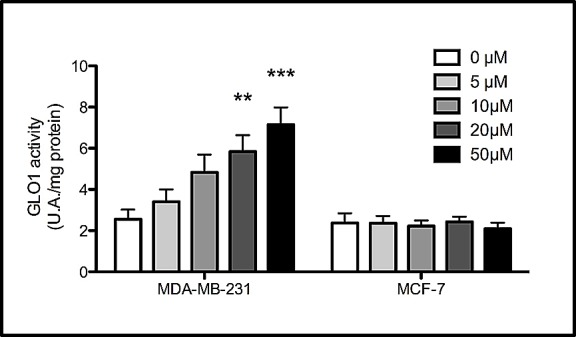
Increased Glo-1 activity in triple negative cells exposed to chronic treatment with MG Glo-1 activity was measured in triple negative MDA-MB-231 and in ER/PR positive MCF-7 cells after three weeks treatment with low doses of MG. A significant increase of Glo-1 activity was observed only in triple negative cells. Results are the average of 4 independent experiments. Data were statistically analyzed with two-way ANOVA followed by Bonferroni multiple comparisons (* *p* ≤ 0.05, **p ≤ 0.01 and ***p ≤ 0.001 vs untreated cells).

To investigate whether triple negative breast cancer cells could respond to MG stimulation by increasing Glo-1 expression and activity, we treated breast cancer cells with MG at 300, 500 or 1000 μM during 6 hours. MG treatment neither affected the level of expression (Figure 6A) nor the activity (Figure 6B) of Glo-1 in MCF-7 and SKBR3 breast cancer cells. However, MG significantly induced a dose-dependent effect on both Glo-1 expression and activity in MDA-MB-231 and MDA-MB-468 triple negative cell lines (Figure 6A and B). Moreover, the 2-way ANOVA analysis proved that the 4 cell lines evaluated responded differently to MG treatment. Indeed, the interaction between MG concentration and cell lines significantly accounted for 32.11% of the total variance (p<0.0001). In order to exclude the potential MG acute toxicity on cancer cells, we also measured Glo-1 activity in MDA-MB-231 and MCF-7 cells after 3 weeks of chronic treatment at lower concentrations of MG (5-50 μM) (Figure 7). As observed under acute treatment, triple negative cells showed a dose dependent induction of Glo-1 activity while MCF-7 cells maintained their basal level of Glo-1 activity in all conditions tested (Figure 7). Moreover, the 2-way ANOVA analysis indicated that the interaction between MG concentration and the 2 cell lines accounted for 20.33% of the total variance (p<0.0015).

## DISCUSSION

While both cancer initiation and progression have been linked to oxidative stress [42] to date there is no clear evidence regarding the importance of the carbonyl stress in such processes. In cancer, reactive oxygen species (ROS) generate genomic damage and instability, activate mitogenic and survival mechanisms and favor cell invasion [43]. However, when the intracellular levels of oxidants reach a cytotoxic threshold, apoptotic and necrotic pathways are activated and cancer cells need to protect themselves from oxidative stress to survive [44].

Beside the well-established oxidative stress concept, endogenous dicarbonyl compounds such as MG also affect proteins, lipids and nucleic acids, leading to AGEs formation and carbonyl stress [45]. The carbonyl stress has been primarily described in diabetes where AGEs accumulation is a major event associated with diabetic complications [46]. High concentrations of MG and formation of AGEs have been reported in diabetic patients [47]. To date, known MG protein targets include albumin, hemoglobin, Sin3A, type IV collagen, alpha A-crystallin, protein p300 and 20S proteasome subunits, all associated with protein dysfunction [45].

Cancer cells predominantly produce energy by increasing their rate of glycolysis and as a consequence, triosephosphates-derived MG is increased in malignant tumor cells. MG is a potent cytotoxic compound and exerts an anti-tumor activity in vivo [48, 49] and was first viewed as a potential therapeutic agent in cancer [50]. However, the recent identification of MG-modified proteins in cancer cells brought the new possibility where MG may have pro-tumorigenic effects. Indeed, MG induced the modification of Hsp-27 at arginine 188 (MG-Hsp27) in cancer cells and MG-Hsp27 facilitated cancer cell evasion from caspases-dependent cell apoptosis [39-41].

Several types of protein modifications by MG have been reported [37, 51], among which those directed to the guanidino group of arginine residues are well represented. In this study, we detected Arg-pyrimidine adducts using immunohistochemistry in a large series of human breast cancer lesions. Non-tumoral breast tissue presented detectable Arg-pyrimidine basal accumulation which was consistently lower than the one observed in matched tumoral samples. We report for the first time the differential accumulation of Arg-pyrimidine moieties between the different breast cancer subtypes analyzed. We observed that triple negative breast cancer tumors had fewer detectable Arg-pyrimidine adducts than the ER/PR positive, HER2 negative and HER2 positive lesions. These original findings were further reinforced by our observation that Arg-pyrimidine adducts were significantly less detectable in triple negative breast cancer cell lines when compared to cell lines from other subtypes. Interestingly, while Arg-pyrimidine immunostaining was mainly cytoplasmic some nuclei presented a positive staining suggesting the presence of nuclear MG-modified proteins in cancer cells. Using the same antibody, Nakadate and collaborators have reported the accumulation of Arg-pyrimidines in the nucleus of neural cells [52]. Ongoing experiments in our laboratory aim to identify new potential nuclear targets of MG such as transcription factors and co-regulator proteins in breast cancer cells.

Glo-1 is the main enzyme involved in the detoxification of MG. It has been reported that Glo-1 expression and activity are increased in breast tumor cells, most likely to control the high MG level spontaneously produced due to high glycolytic activity [33]. Glo-1 expression was significantly higher in Her-2 positive breast tumors when compared with Her-2 negative ones. Thus, Her-2 positive tumors associated with an aggressive phenotype and a poor prognosis overexpress Glo-1 [53]. However, Glo-1 enzymatic activity was not evaluated in these tumors. In this study, Glo-1 immunostaining performed on human triple negative and triple positive breast cancer lesions did not show any significant difference. Conversely, the measurement of Glo-1 activity performed on fresh samples showed a significant increase in Glo-1 enzymatic activity in triple negative tumors. Accordingly, we found that MDA-MB-231 and MDA-MB-468 triple negative breast cancer cells adapted their level of Glo-1 expression and activity when treated with exogenous increasing doses of MG. Considering that MCF-7 cells remained stable for Glo-1 under the same conditions, it is tempting to speculate that triple negative breast cancer cells increased their Glo-1 activity as a control defense mechanism to prevent MG accumulation. This hypothesis was further sustained by the increased dose-dependent Glo-1 activity observed in both MDA-MB-231 and MDA-MB-468 cells in response to a chronic sub-toxic MG treatment when compared to MCF-7 cells. Such adaptive response to MG has been previously reported in Carl-1 human melanoma cells and was described as a defense mechanism against MG toxicity [54].

Our study adds to the growing body of evidence suggesting a major role of non-enzymatic glycation in cancer. The increase of Glo-1 activity may represent a strategy adopted by aggressive cancer cells such as triple negative breast cancer cells as a defense mechanism against the glycation damage induced by high intracellular MG accumulation. Our data suggest that inhibition of Glo-1 activity in triple negative breast tumors could be a new potential therapeutic strategy. In support of this concept, previous studies reported that a specific Glo-1 inhibitor, S-p-bromobenzyl-glutathione cyclopentyl diester (SpBrGSHCp_2_), demonstrates an anti-tumor effect against Glo-1-overexpressing tumors that are unresponsive to conventional therapies. This Glo-1 inhibitor has been shown to increase the sensitivity of human leukemia cells to anti-tumor agents [55]. In human lung cells with high expression and activity of Glo-1, the treatment with SpBrGSHCp_2_ activated apoptosis through the activation of the stress-activated protein kinases JNK1 and p38 MAPK which finally led to caspase activation [32]. In human prostate cancer cells, the treatment with SpBrGSHCp_2_ significantly affected tumor growth [14].

Oxidative stress has several pro-tumorigenic effects but it also exerts an anti-tumorigenic action as it has been linked to senescence and apoptosis. As a major actor of the carbonyl stress, MG also plays a dual role as a cytotoxic compound and as a pro-tumorigenic factor depending on the global cellular context. Moreover, one must consider that oxidative and carbonyl stresses are tightly linked together and may work in tandem to influence tumor phenotype. Indeed, the formation of both ROS and AGEs by cancer cells generates a sequence of reactions mutually enhancing each other [56].

Our study opens a new alley of investigations toward individualized cancer therapy based on the determination of metabolomic changes in tumors that might enable the targeted blockade of cancer progression.

## MATERIALS AND METHODS

### Clinical tumor samples

Patient tissue samples were obtained from the Pathology Department of the University Hospital of Liège in accordance with ethical guidelines of the University of Liège (Liège, Belgium). The immunohistochemical analysis was conducted on a collection of 93 breast cancer patients grouped into 4 biological subtypes (triple negative, triple positive, HER2− and HER2+) and 2 normal breast specimens obtained from reduction mammoplasties. ER, PR, and HER2 status was evaluated routinely by anatomo-pathological examination. In this series, HER2 positive tumors were all negative for ER and PR receptors and all HER2 negative tumors were positive for both hormone receptors. Patients received standard guideline-based according to standard guideline: hormonotherapy for all patients with ER-positive tumours, trastuzumab for those with HER2-positive tumours, anthracycline chemotherapy for high-risk lymph node-negative lesions, and anthracycline plus taxane chemotherapy for lymph node positive tumors. Western blot analysis was conducted on total protein extracts from 7 breast ductal adenocarcinoma (grade II and III) and their matched non-tumoral counterpart.

### Cell lines

Human breast cancer cell lines BT549, Hs578T, MCF-7, MDA-MB-231, SKBR3 were obtained from the American Type Culture Collection (ATCC) and MDA-MB-468 cell line was a kind gift from Prof. Sebastiano Andò (Laboratory of General Pathology, University of Calabria, Italy). Breast cancer cell lines usually cultured in DMEM (standard glucose concentration of 4.5 g/L, Lonza) containing 10% fetal bovine serum, and 2 mM L-glutamine, were adapted to grow in DMEM medium with a glucose concentration of 1 g/L for several weeks. This glucose level is physiological and reflects the in vivo concentration in human serum. Conversely, the routine culture media concentration of 4.5 g/L corresponded to a diabetic condition.

### Western blot analysis and antibodies

Cell and tissue samples were extracted in RIPA buffer (150 mM NaCl, 0.5% Na-deoxycholate, 1% Triton X-100, 0.1%SDS, 50 mM Tris-HCl pH 7.5) containing protease and phosphatase inhibitors (Roche). After incubation under rotation at 4°C for 40 minutes, lysates were then centrifugated at 14,000 g for 15 minutes at 4°C to remove insoluble debris. Protein concentrations were determined using the BCA assay (Pierce). Protein extracts were then separated by SDS-PAGE and transferred to a PVDF membrane. Next, the membranes were blocked for 1h in TBS-Tween containing 5% nonfat dried milk (Bio-Rad) and incubated with the primary antibodies overnight at 4°C. The membranes were probed with anti-Arg-pyrimidine (mAb6B) monoclonal antibody (1:10,000). The specificity of this antibody has been previously confirmed by competitive ELISA and it has been shown to not react with other MG-arginine adducts such as 5-hydro-5-methylimidazolone and tetrahydropyrimidine [57]. Anti-Human Glyoxalase I monoclonal antibody (1:1,000 dilution, cat#02-14, BioMac Leipzig) and anti-beta-actin (1:5,000 dilution, cat# A5441, Sigma) were used. Horseradish peroxidase-conjugated secondary antibodies [anti-mouse, 1:6,000 dilution (Dako) or anti-rabbit 1:3,000 (Invitrogen)] were used to visualize bound primary antibodies, with the ECL Western blotting substrate (Pierce). Where indicated, ImageJ software 1.46r (imagej.nih.gov) was used for semi-quantitation using beta-actin as a loading control.

### Immunohistochemistry on breast tissues

Formalin-fixed paraffin-embedded sections were deparaffinized in xylene and rehydrated. To block endogenous peroxidase activity, the tissues were treated with 3% hydrogen peroxide in methanol for 30 minutes and washed in PBS for 20 minutes. Antigen retrieval was performed in 10 mM sodium citrate, pH 6.0 for 40 minutes at 95°C. Sections were then incubated with 1.5% normal horse serum (cat#S-2000, Vector Laboratories) for 30 minutes to block the nonspecific serum-binding sites. Then, sections were incubated with anti-Arg-pyrimidine (1:2,000 dilution) or anti-human Glyoxalase I (1:100 dilution) antibody overnight at 4°C. Antibody binding was detected using an anti-mouse biotinylated secondary (cat#BA-2000, Vector Laboratories) for 30 minutes followed by incubation with the avidin-biotin-peroxidase complex (Vectastain ABC Kit, Vector Laboratories). Immunoreactivity was revealed using 3,3′ diaminobenzidine tetrahydrochloride (DAB). The slides were counterstained with hematoxylin, dehydrated and mounted.

### Evaluation of immunohistochemical staining

The immunohistochemically stained sections were reviewed by two examiners including an anatomopathologist (E.B). Scoring of the staining was done according to the intensity of the staining (0, 1+, 2+, 3+) and the percentage of positive cancer cells (0-25%, 25-50%, 50-75%, 75-100%). The results obtained with the 2 scales were multiplied together as we previously described [58], yielding a single scale with steps of 0, 1+, 2+, 3+, 4+, 6+ and 9+ where 0, 1+ and 2+ were considered to be negative or weak staining and 3+, 4+, 6+ and 9+ were considered to be medium or strong staining.

### Glyoxalase I assay

The activity of Glo-1 was measured in breast cancer cell lines and in human breast cancer tissues after protein extraction in RIPA buffer, by measuring the initial rate of S-D-lactoylglutathione formation from the hemimercaptal obtained by preincubation of an equimolar (1 mM) mixture of MG (cat#M0252, Sigma) and GSH (cat#G4251, Sigma) in 50 mM sodium phosphate buffer, pH 6.8, at 25°C for 15 minutes. S-D-lactoylglutathione formation was followed spectrophotometrically by the increase in absorbance at 240 nm at 25 °C. One enzyme unit was defined as the amount of enzyme that catalyzes the formation of 1 μmol of S-D-lactoylglutathione per minute at the saturating substrate concentration.

Chronic treatment with MG. Breast cancer cells MDA-MB-231 and MCF-7 were treated with MG at different concentrations (5, 10, 20, 50 μM) for three weeks. The first week, a daily treatment was performed and for the remaining 2 weeks the treatment was repeated twice a week. Glo-1 activity was measured as described above on cell lysates after protein extraction in RIPA buffer.

### Statistical analysis

The data were statistically analyzed using either one-tailed Student t-test or with two-way ANOVA followed by Bonferroni multiple comparisons. One-tailed t-test was selected because the H1 hypothesis was “triple negative Glo-1 activity is below or equal to triple positive”. Number of cases for Arg-pyrimidine or Glo-1 staining in each cancer subtypes was analyzed in a contingency table using chi-square test and Fisher's exact test, respectively. P values less than 0.05 were considered statistically significant.
